# Understanding what matters most to people with multiple myeloma: a qualitative study of views on quality of life

**DOI:** 10.1186/1471-2407-14-496

**Published:** 2014-07-09

**Authors:** Thomas R Osborne, Christina Ramsenthaler, Susanne de Wolf-Linder, Stephen A Schey, Richard J Siegert, Polly M Edmonds, Irene J Higginson

**Affiliations:** 1 Department of Palliative Care, Policy and Rehabilitation, King’s College London, London, UK; 2 Department of Haematological Medicine, King’s College Hospital and King’s College London, London, UK; 3 School of Public Health and Psychosocial Studies and School of Rehabilitation and Occupational Studies, Auckland University of Technology, Auckland, New Zealand; 4 Department of Palliative Care, King’s College Hospital, London, UK

**Keywords:** Cancer, Oncology, Haematology, Multiple myeloma, Quality of life, Outcome assessment

## Abstract

**Background:**

Multiple myeloma is an incurable haematological cancer that affects physical, psychological and social domains of quality of life (QOL). Treatment decisions are increasingly guided by QOL issues, creating a need to monitor QOL within clinical practice. The development of myeloma-specific QOL questionnaires has been limited by a paucity of research to fully characterise QOL in this group. Aims of the present study are to (1) explore the issues important to QOL from the perspective of people with multiple myeloma, and (2) explore the views of patients and clinical staff on existing QOL questionnaires and their use in clinical practice.

**Methods:**

The *‘Issues Interviews’* were semi-structured qualitative interviews to explore the issues important to QOL in a purposive sample of myeloma patients (n = 20). The ‘*Questionnaire Interviews’* were semi-structured qualitative interviews in a separate purposive sample of myeloma patients (n = 20) to explore views on existing QOL questionnaires and their clinical use. Two patient focus groups (n = 7, n = 4) and a focus group of clinical staff (n = 6) complemented the semi-structured interviews. Thematic content analysis resulted in the development of a theoretical model of QOL in myeloma.

**Results:**

Main themes important to QOL were Biological Status, Treatment Factors, Symptoms Status, Activity & Participation, Emotional Status, Support Factors, Expectations, Adaptation & Coping and Spirituality. Symptoms had an indirect effect on QOL, only affecting overall QOL if they impacted upon Activity & Participation, Emotional Status or Support Factors. This indirect relationship has implications for the design of QOL questionnaires, which often focus on symptom status. Health-service factors emerged as important but are often absent from QOL questionnaires. Sexual function was important to patients and difficult for clinicians to discuss, so inclusion in clinical QOL tools may flag hidden problems and facilitate better care. Patients and staff expressed preferences for questionnaires to be no more than 2 pages long and to include a mixture of structured and open questions to focus the goals of care on what is most important to patients.

**Conclusion:**

Existing QOL questionnaires developed and validated for use in myeloma do not capture all that is important to patients and may not be well suited to clinical use.

## Background

Multiple myeloma is a malignant proliferation of plasma cells affecting 0.4 to 5 per 100,000 individuals per year globally, with a higher incidence in developed countries and slowly increasing incidence worldwide [1]. The pattern of end-organ damage is complex, including destruction of the bones, bone marrow failure and renal failure, leading to impairments in physical, psychological and social domains of quality of life (QOL) [2-4]. Whilst myeloma remains incurable the survival of patients has significantly improved in the course of the last 15 years as a result of increased availability of more active and less toxic drugs [5]. However, toxicity remains an issue and patients are living longer with complications of their disease and side effects of treatment. New drug trials and treatment decisions are therefore increasingly dictated by considerations around QOL.

A number of models of QOL have been reported throughout the literature. These include those based on human need [6]; expectations [7]; functioning, disability and health [8]; personal characteristics as mediators [9]; and phenomenological models based on individual perceptions [10]. A model of QOL for use in healthcare was proposed by Wilson and Cleary [11], which provides a taxonomy of five levels of heath outcome for use across different diagnoses: biological and physiological factors; symptoms; functioning; general health perceptions; and overall QOL. Wilson and Cleary linked familiar biological variables to overall QOL, to demonstrate how clinical interventions might be better designed to improve QOL, through modification of the intervening variables. A recent systematic review found Wilson and Cleary’s model to be the most widely used in the literature [12].

There are a number of QOL questionnaires validated for use in myeloma [13], but only two myeloma-specific tools have been developed. The European Organisation for Research and Treatment of Cancer core cancer questionnaire (EORTC-QLQ-C30) and its myeloma-specific module (MY20) are the most comprehensively validated and were designed predominantly as research tools [13-16]. The only other myeloma-specific QOL questionnaire is the recently developed Functional Assessment of Cancer Therapy – Multiple Myeloma (FACT-MM), although its item pool was developed with limited patient involvement and its authors call for further development work using larger and more heterogeneous patient samples [17].

Assessment of QOL is important in both research and clinical practice. Alongside clinical care, QOL assessment can monitor response to treatment, focus goals of care, and facilitate communication [18-21]. Several authors have recommended that QOL assessment should form part of the routine care of myeloma patients [2,3,22], yet existing QOL questionnaires may not be well suited for this purpose, and may not capture all the issues important to patients [13]. Qualitative enquiry is a vital step to ensure good content validity during the development of QOL questionnaires [23,24]. There are some qualitative studies looking at the experience of myeloma from the patient’s perspective. These have explored lived experience [25-28], trauma and post-traumatic growth [29] and distress [30], but there is a paucity of qualitative research to directly characterise the meaning of QOL in this group [13].

This study is part of a research programme to investigate ways to improve QOL assessment in clinical practice. The aims of the current study are (1) to explore the issues important to QOL from the perspective of people diagnosed with multiple myeloma, and (2) to explore the views of patients and clinical staff on existing QOL questionnaires and their use in clinical practice.

## Methods

### Overview of study design

This study is reported in accordance with the RATS guidelines for qualitative research [31]. The study used a combination of semi-structured qualitative interviews and focus groups to address the overall aims. The ‘*Issues Interviews’* were semi-structured interviews of myeloma patients (n = 20) to explore the issues important to QOL from the patients’ perspective. The ‘*Questionnaire Interviews’* were semi-structured interviews in a separate sample of myeloma patients (n = 20) to explore views on existing QOL questionnaires in terms of content, design and preferences for clinical use. The semi-structured interviews were complemented by two patient focus groups (n = 7, n = 4). Each patient focus group was split in two halves to mirror the Issues Interviews and Questionnaire Interviews – the first half of each focus group explored issues important to QOL, and the second half explored views on existing QOL questionnaires. Finally, a focus group of clinical staff (n = 6) explored views on existing QOL questionnaires, and the desired clinical utility of such tools in routine practice.

The combined use of individual interviews and focus groups is recommended best practice for establishing content validity in both new and existing patient reported outcome measures [23]. The combined use of these methods helps confirm the validity of results through triangulation [32]. In the present study, individual interviews were carried out to allow greater depth of probing and offer privacy for the discussion of sensitive QOL issues that may be hard to raise in a group setting (such as psychological problems or sexual function). Focus groups were also carried out since group discussion may ignite different memories and ideas for individual participants, with new or additional themes emerging from group interaction. For these reasons, the two interview methods were considered to be required and to complement one another, even though the same questions were asked of participants within the two different interview types.

### Participants and setting

Participants were recruited from both inpatient and outpatient settings across three organisations in inner London. The lead site was King’s College Hospital NHS Foundation Trust, with additional participants recruited from Guy’s and St Thomas’ NHS Foundation Trust and St Christopher’s Hospice to ensure a balance from different clinical settings and stages of disease. King’s College and Guy’s and St Thomas’ Hospital Trusts provide tertiary haemato-oncology services to London and south-east England, and King’s contains the largest bone marrow transplant centre in Europe. St Christopher’s is one of the UK’s largest hospices, offering inpatient and community hospice care to people across 5 boroughs in south-east London.

Patients could participate in only one interview or focus group across the whole study. Inclusion criteria for patients were age 18 years or older; confirmed diagnosis of multiple myeloma; having been told the diagnosis; and capacity to give written informed consent. Exclusion criteria for patients were those too unwell, symptomatic or distressed to participate (as judged by the clinical team); severe neutropenia where contact with researcher may pose a risk; unable to understand written and spoken English; and those for whom myeloma was not the most important health problem (as judged by the patient). Inclusion criteria for clinical staff were those with at least 2 years’ experience working regularly with myeloma patients in the specialist haematology setting.

During recruitment all potential participants were screened by a member of their clinical team to assess eligibility before being approached about the study. Following the initial approach, potential participants were offered at least 24 hours to consider if they wanted to take part. Participation was voluntary and interviews took place at a time and place convenient to participants (hospital, home or other location requested by them), except for focus groups which took place on the hospital site.

Recruitment was carried out by a team of three research staff from the Department of Palliative Care, Policy and Rehabilitation at King’s College London (TRO, CR and SdW). TRO was a clinical research fellow with a background as a medical doctor in both haematology and palliative medicine, CR was a research assistant with a background in psychology, and SdW was a research nurse with a background in oncology and palliative care nursing. The research team was distinct from the clinical team – none of the researchers were involved in the clinical care of study participants. The research team introduced themselves to patients as ‘researchers’ to avoid any potential coercion when deciding whether or not to participate, and reduce any potential bias in study participants’ responses during the interviews.

### Sampling

Both the Issues Interviews and Questionnaire Interviews used purposive sampling by gender, age (<65 or >65), ECOG performance status (0–2 or 3–4) and disease phase (newly diagnosed, plateau, or relapsed) [33]. These criteria were chosen to achieve maximum variation across key characteristics thought to potentially influence QOL, based on existing literature. Sample size was not fixed at the start, but was based on data saturation and the purposive criteria. Data saturation was defined as two consecutive interviews with no new emergent themes or issues important to QOL, and was recorded using saturation tables as recommended in the development of patient reported outcomes research [34].

For the patient focus groups convenience sampling was used. There were many practical difficulties in gathering patients together as a group, with rapidly changing treatment plans and people unable to travel long distances. The number of patient focus groups was not decided at the outset, but was continued until no new QOL issues or themes emerged from two consecutive groups. For the clinician focus group a purposive sample was used to ensure a balance of different professional groups and levels of seniority (senior and junior doctors; senior and junior nurses; allied health professionals). Only one focus group of clinical staff was carried out due to the practical challenges of gathering a multidisciplinary group together.

### Issues interviews

These took place in a private room with only the interviewer and participant present to help reduce any potential bias in the participants’ responses. The interview topic guide was split into two halves, covering (i) what issues are important to QOL; and (ii) what things impact on QOL. In practice, these two halves were not distinct, and the course of the interview was guided by the participant’s responses. All interviews began with open questions, with leading questions avoided throughout. Participants were probed about specific QOL issues only if they were raised in response to an open question. This meant the discussion was based only on the issues important to participants.

The Issues Interviews were all conducted by a single researcher (TRO). Interviews were audio recorded and transcribed verbatim. Transcription was split between TRO and a professional transcriber. All professional transcripts were read by TRO alongside the recording to check for accuracy prior to analysis.

### Questionnaire interviews

Each participant was asked to complete four QOL questionnaires. These were EORTC-QLQ-C30, its myeloma module (MY24), the Palliative care Outcome Scale (POS) and a single item global QOL question using a visual analogue scale from 0 to 100.

The EORTC-QLQ-C30 and MY24 are 30- and 24-item tools respectively, which together form a 54-item tool. These were chosen as the most comprehensively validated tools in patients with myeloma [13,15,16]. The MY24 module has been revised to the MY20 by removal of 4 items by the EORTC group, who currently recommend the revised MY20 for use. We chose to use the MY24 at the outset of the current study, since the 4 items were removed due to poor psychometric performance (ceiling effects), rather than a belief that they are not important to QOL [14]. It was therefore considered important to assess participants’ views towards the QOL issues covered by the 4 removed items.

The POS was added as a comparator since it is shorter (12 items) and was designed for clinical use (as opposed to the EORTC tools, which were primarily designed for use in research settings). A systematic review of existing QOL questionnaires validated in myeloma did not identify any tools specifically designed for clinical use [13], so the POS was selected to allow exploration of participants’ views on such tools. The POS was developed for use in people with palliative care needs irrespective of diagnosis or clinical setting, and is suitable for use in those diagnosed with chronic or progressive diseases [35,36], making it appropriate for use by myeloma patients.

The EORTC and POS questionnaires contain a mixture of numerical and Likert scales, with a single open question in the POS. The single item visual analogue scale was therefore added to allow preferences to be explored around this additional type of scaling.

Participants completed and were asked about each questionnaire in turn. They were asked if each item was important or relevant to their QOL and about the most important items, missing items (important QOL issues not covered), acceptable questionnaire length for use in routine clinical practice and preferences for layout, scaling and response options. The Questionnaire Interviews were completed by one of three researchers (TRO, CR or SdW). They were not transcribed but were analysed directly from the audio recordings [37].

### Focus groups

All focus groups were chaired by TRO and were audio recorded. Participants were identified by name so the data could be analysed at the level of the individual. Patient focus groups were each split into two halves, the first half mirroring the Issues Interviews and the second half mirroring the Questionnaire Interviews. There was a break in between the two halves during which the participants completed the same set of questionnaires as for the Questionnaire Interviews.

The focus group of clinical staff explored views towards the same set of QOL questionnaires shown to patients in the Questionnaire Interviews. This focus group explored perspectives on routine clinical use, preferred utility of QOL tools in clinical practice, most clinically relevant questions, preferred layout, presentation and response formats. The topic guide used the guiding questions suggested elsewhere [38]: (i) What are the issues frequently discussed by myeloma patients and clinical staff; (ii) are there important issues less frequently addressed; (iii) can these issues be addressed using questionnaires; (iv) what is the clinical value of QOL questionnaires; and (v) suggestions for improving existing QOL questionnaires to make them more useful in clinical practice. The focus group of clinical staff was analysed directly from the audio recording.

### Analysis

The Issues Interviews and corresponding half of each patient focus group were analysed together. They were transcribed verbatim, imported into NVivo software and analysed using thematic content analysis [39,40]. Data analysis took place alongside recruitment so emergent themes could guide sampling and probing in future interviews. The first transcripts were analysed and an initial coding frame was developed by TRO. To reduce bias, a second researcher (CR) double-coded the complete set of transcripts. Both TRO and CR coded all transcripts alongside recruitment and met regularly to discuss any refinements to the coding frame as the study progressed. Once recruitment was complete and the coding frame finalised, TRO re-coded the complete set of transcripts according to the final coding frame. CR then re-coded a subset (10%) using the final coding frame, to check for consistency. Discrepancies were resolved by consensus.

The Questionnaire Interviews, corresponding half of each patient focus group, and the professional focus group were all analysed together. Data were extracted directly from the audio into tables, constructed with participant numbers across the top, and questionnaire items/attributes listed down the left hand column. Examples of attributes of interest were font size, questionnaire length, response options and scaling, recall period, most important questions, missing items or gaps, other comments. After the table was populated analysis could take place ‘by-item’ or ‘by-attribute’ where all views could be considered in aggregate. Data were extracted into tables by TRO and CR, with further analysis/aggregation of the data carried out by TRO.

Throughout this process TRO developed a theoretical model of QOL based on the issues and themes emerging from the data. The model was developed by taking account of but not being restrained by existing theory and models of QOL from the literature. It was not clear at the outset if an existing model would be modified or if a completely new model would be required, but it was considered important to take account of existing work and build on this where appropriate, rather than ‘re-inventing the wheel’. The model mainly drew on data from the Issues Interviews and corresponding half or each focus group, but did also incorporate some data from the Questionnaire Interviews (e.g. views about missing questionnaire items important to QOL). The model was regularly discussed with the wider project steering group throughout its development, comprising three junior researchers with backgrounds in medicine (TRO), psychology (CR) and nursing (SdW), and a professor of haematology (SAS) and professor of palliative care (IJH).

### Ethical issues

Research Ethics Committee approval was granted by the South East London REC-3 (ref 10/H0808/133). All participants gave written informed consent to take part, including for their anonymised views to be shared in scientific publications and meetings. Care was taken to avoid identifiable information in quotations used in the manuscript.

## Results

### Participants

74 eligible patients were approached and 51 agreed to participate (45 from King’s College Hospital, 4 from St Christopher’s Hospice and 2 from Guy’s Hospital). The majority of participants were recruited from King’s College Hospital due to earlier Research and Development approval to recruit from this site. Reasons for declining were a reluctance to share personal experiences with others (7), feeling too unwell (4), being too busy (3), previous bad experiences with research (1), living too far away (1), and no reason given (7). Characteristics of recruited patients are shown in Table 1.

**Table 1:** Sample Characteristics (patients, n = 51)

	**Study components**			**Total (n = 51)**
	**Issues interviews (n = 20)**	**Questionnaire interviews (n = 20)**	**Patient focus groups (n = 11)**	
**Gender**				
Male	10	12	8	30
Female	10	8	3	21
**Age**				
Median (range)	66 (41–78)	63.5 (46–81)	60 (41–70)	64 (41–81)
<65	10	12	6	27
≥65	10	8	5	24
**Marital status**				
Single	0	2	1	3
Married/partnered	14	15	9	38
Divorced/separated	3	2	1	6
Widowed	3	1	0	4
**Ethnicity**				
White British	13	12	9	34
White Other	1	1	0	2
Black African/Caribbean	5	5	2	12
Other	1	2	0	3
**Religion**				
Atheist	4	0	0	4
Christian	16	17	11	44
Other	0	3	0	3
**Highest educational level**				
Did not finish school	3	0	3	6
Secondary school graduate	5	9	3	17
College/technical qualification	9	9	2	20
First degree	2	1	3	6
Higher degree	1	1	0	2
**Occupation status**				
Working or student	3	5	1	9
Not working	3	3	2	8
Retired	14	12	8	34
**ECOG performance status**				
0-2	10	11	11	32
3-4	10	9	0	19
**Disease phase**				
Newly diagnosed	7	6	0	13
Stable/plateau phase	7	7	9	23
Relapsed/progressive	6	7	2	15
**Currently on treatment**				
Yes	7	13	3	23
No	13	7	8	28
**Months since diagnosis**				
0-12	8	8	1	17
13-24	7	1	1	9
25-36	2	0	0	2
37-48	1	4	1	6
Over 48	2	7	8	17

9 clinical staff were approached (all from King’s College Hospital), and 6 agreed to participate. All staff who declined did so due to clinical commitments. Characteristics of recruited staff are shown in Table 2.

**Table 2:** Sample Characteristics (clinical staff, n = 6)

**Gender**	
Male	2
Female	4
**Age**	
Median (range)	37 (32–61)
**Time since qualification**	
Years in clinical practice: Median (range)	11 (5–38)
Years in haematology: Median (range)	8.5 (3–30)
**Profession group and grade**	
Medical: Haematology consultant (myeloma specialist)	1
Medical: Haematology junior doctor (specialist registrar)	1
Nursing: Myeloma clinical nurse specialist	1
Nursing: Haematology ward based	2
Allied health: Haematology specialist physiotherapist	1

Participants were recruited over a 20-month period. The Issues Interviews recruited over months 1–16, Questionnaire Interviews over months 11–20; and focus groups over months 14–19. The mean length of the Issues Interviews was 52 minutes, Questionnaire Interviews 73 minutes, and focus groups 117 minutes.

For the Issues Interviews theoretical saturation was reached after 14 participants. However, only 5 of the first 14 participants had an ECOG performance of 3–4, and early analysis revealed that functional status may be an important determinant of QOL. Therefore recruitment was continued to target more participants with poor performance status. Two new issues emerged in the 18th interview, and recruitment was stopped after 20 participants. The Questionnaire Interviews yielded no additional QOL issues, so recruitment was continued to 20 interviews once a balance of the purposive criteria had been reached. No new QOL issues emerged in the patient focus groups so recruitment was stopped after two groups.

### Issues important to QOL

**Figure 1:**
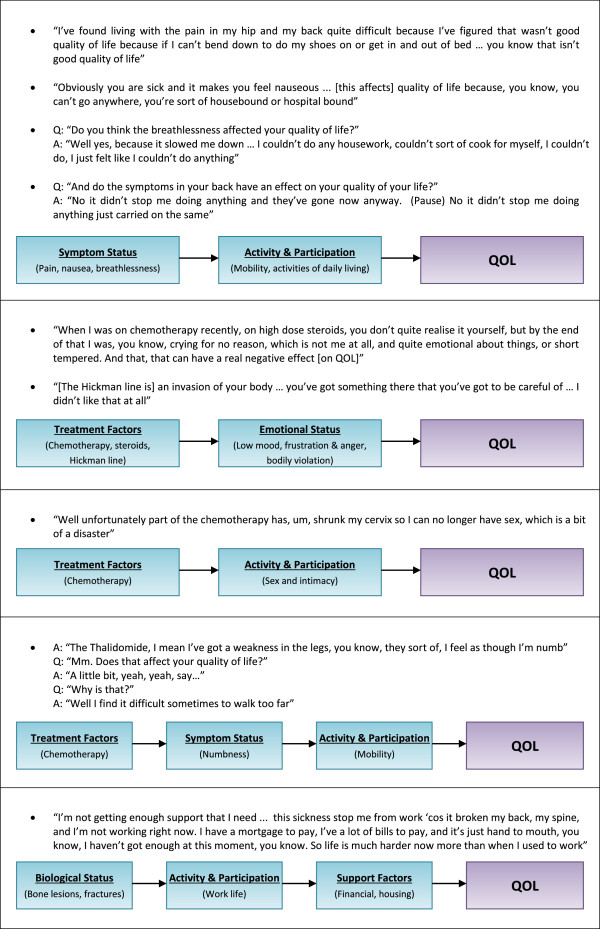
The relationship of biological status, treatment factors, symptom status, emotional status, activity & participation, and support factors to overall QOL.

The term ‘QOL’ was understood by all participants, with none asking for clarification of its meaning. During the Issues Interviews (and corresponding half of each focus group) health-related issues dominated the discussion in most cases. This was true even though more general questions were asked *(“What things are important to your QOL?”).* Participants raised a total of 80 issues that were important or affecting QOL. These were grouped into 9 main themes: Biological Status, Treatment Factors, Symptom Status, Activity & Participation, Emotional Status, Support Factors, Expectations, Adaptation & Coping and Spirituality. Examples from the data showing how each theme relates to overall QOL are shown in Figure 1.

The themes most closely related to QOL were Emotional Status, Activity & Participation and Support Factors. A change in any of these seemed to lead directly to a change in QOL. Rather than *impacting* on QOL, these themes seemed to form the essence of QOL itself (Figure 1). Within Support Factors, there was a particularly strong role for health-service factors. Every participant in the Issues Interviews mentioned some property of the health-service as being important to their QOL, and this sometimes dominated the discussion.

The themes of Biological Status, Treatment Factors and Symptoms Status were important to QOL, but less closely related. These themes were often raised by participants, but further probing revealed that they did not *necessarily* affect QOL. Biological Status, Treatment Factors and Symptoms Status only affected QOL if they affected one of the more fundamental themes ‘closer’ to QOL (Emotional Status, Activity & Participation or Support Factors) (Figure 1).

**Figure 2:**
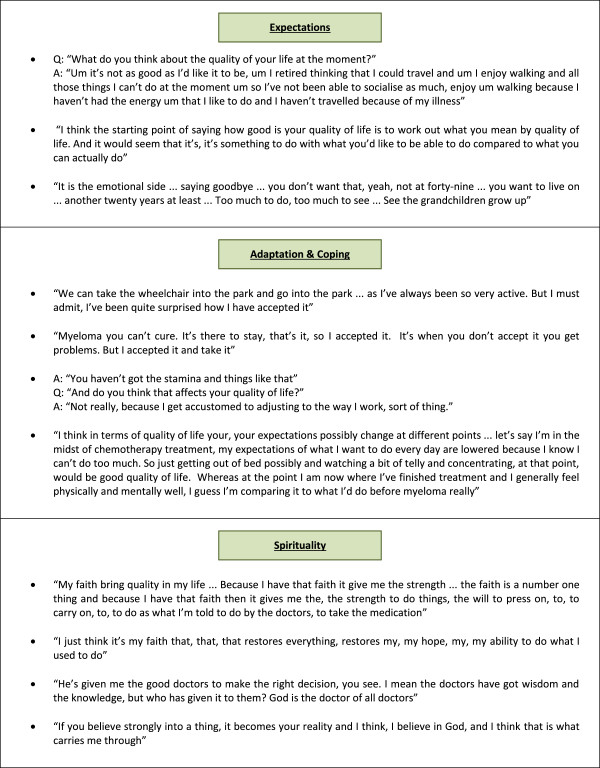
The role of expectations, spirituality, adaptation and coping in determining QOL.

Expectations, Adaptation & Coping and Spirituality had a more complex relationship to QOL. These were personal characteristics that governed how much a problem affected QOL for a given individual. Examples from the data are shown in Figure 2. Taking the first example under Expectations (Figure 2), this participant reported a reduction in QOL because the illness was preventing him from walking, travelling and socialising, but the impact of these functional impairments on QOL seemed to be driven partly by the individual’s *expectation* that these activities would be possible during their retirement. By contrast, some participants described problems or impairments that had *not* affected their QOL, because they had managed to adapt to live life differently (see examples under Adaptation & Coping, Figure 2). For some participants it was their faith in God that provided the strength and hope needed to cope with their illness, or provide support in the form of doctors, wisdom and knowledge. Faith in God acted for some participants like a lens through which all experience was viewed, with examples shown under the theme of Spirituality (Figure 2).

### Development of the theoretical model of QOL

**Figure 3:**
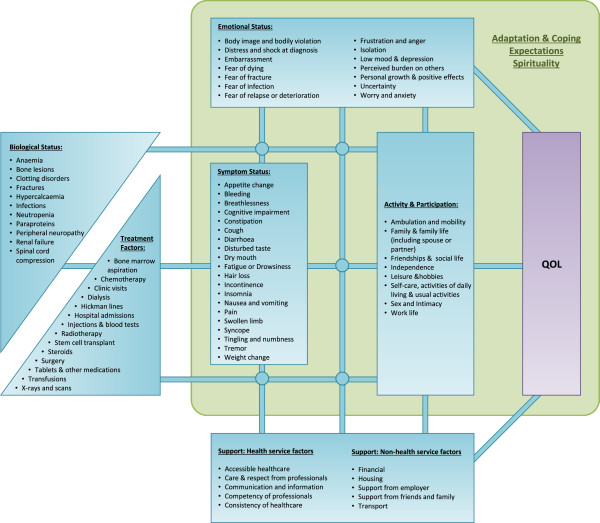
**Theoretical model of the QOL of people with multiple myeloma. Adapted from Wilson and Cleary**[11]**.**

The theoretical model of OQL developed in the present study sought to represent the relationships between overall QOL and the themes identified above. The model of QOL proposed by Wilson and Cleary [11] was adapted, since it was designed to link clinical variables to QOL and seemed a good fit for many of the themes emerging from the data. The adapted theoretical model of QOL in myeloma is shown in Figure 1. The boxes in the model correspond to the themes identified above, and the lists within each box show the issues raised by participants during the interviews and focus groups.The lines linking the boxes/themes represent reciprocal causal relationships, acting in both directions. A change in Biological Status may cause a change Symptoms Status (e.g. fractures causing pain), but the converse may also be true and a change in Symptoms Status may cause a change Biological Status (e.g. vomiting causing renal failure). The intersections of the lines in the model create junctions where causal relationships can travel in any direction – representing the interconnectedness of the themes. For example, a change in Treatment Factors might affect Emotional Status, Symptom Status and Activity and Participation (chemotherapy causing mood swings, peripheral numbness and impaired sexual function – Figures 1 and 3).The boxes/themes for Emotional Status, Activity & Participation and Support Factors have direct connections to QOL, but this is not the case for Biological Status, Treatment Factors and Symptoms Status. This signifies that changes in Emotional Status, Activity & Participation and Support Factors led directly to changes in QOL, whereas changes in Biological Status, Treatment Factors and Symptoms Status only affected QOL if one of the three more fundamental themes ‘closer’ to QOL was also affected (Figures 1 and 3).The overarching box represents the themes of Adaptations & Coping, Expectations and Spirituality. These had a more complex relationship to overall QOL, acting to mediate the causal relationships between themes. For example, the degree to which reduced mobility affected QOL being determined by the individual’s expectations, illness adaption, and spiritual beliefs (Figures 2 and 3).

### Views on existing QOL questionnaires

During the Questionnaire Interviews (and corresponding half of each focus group) participants often found it difficult to identify the most important items, since they found it difficult to consider so many questions in aggregate. Out of 31 participants who were shown the questionnaires, only 6 were able to state the most important items. Out of these 6, most participants highlighted more than one item as important, and the same issues tended to recur. The items raised as important were mobility (5 participants), pain (3 participants), healthcare (2 participants) and dying (1 participant). Similar difficulties were faced when asking participants to highlight any missing issues, with only 9 participants giving a response. Again, the same issues tended to recur: healthcare (5 participants), information about the future (3 participants), sexual function (3 participants) and independence (2 participants).

Participants commented that some questions in existing tools were vague and hard to interpret (e.g. “Did you need to rest?” and “Have you felt ill?”); and some words were hard to understand (“Have you felt nauseated?”). A number of participants complained that questions appeared to overlap in their meaning, (“Did you need to rest?/Were you tired?”, “Did you worry/Did you feel tense?”). Acceptability of existing tools was reduced where there was perceived repetition, for example multiple questions about pain.

Participants were asked about the ideal length for a questionnaire. This was intended to be a question about the acceptable number of items, but participants usually gave their response in terms of the number of pages or amount of text to read. Some participants noted that the EORTC tools felt less burdensome than the POS because the response options were the same for multiple items in the EORTC, whereas the POS has different response options for each question. This meant that completion of the EORTC involved less reading, even though it contains more items. Participants framed their preferences in terms of the amount of text/reading and preferred tools that extended to no more than 2 sides of A4 paper.

There were mixed views on the best type of scaling (Likert vs. numerical scales), although there was a dislike of the visual analogue scale by all participants due to difficulty understanding how to complete it and generally needing more explanation and prompting. The EORTC asks the respondent to consider the past week, whereas the POS asks about the past 3 days. Many participants had no preference about this. All those expressing a preference preferred 1 week or longer. Recall was easier over the past week than over 3 days. Some participants noted that questions about healthcare or relationships with doctors did not make sense over a fixed time frame, and were something that evolved over the whole of their illness experience. All participants supported the inclusion of an open question (as found in the POS), to allow them to raise issues not covered elsewhere in the questionnaire.

### Focus group of clinical staff

The focus group of clinical staff identified a number of desired uses for QOL tools in clinical practice. These were to identify and prioritise problems from the patient’s perspective, mitigate against time pressure in busy clinical settings, facilitate discussion of embarrassing issues, compare QOL to before treatment started, trigger referral to other services, and to aggregate data for clinical audit and allocation of resources:

*"Do all the people we treat in a certain way end up with a particular problem? … if it is, then we've got to do something about it”* (Haematology doctor)

The most clinically useful questions from the professionals’ point of view were about anxiety, depression, pain, fatigue, and sexual function. There was consensus that sexual function is an important problem in myeloma, but is often hard for patients and clinicians to discuss and might therefore be useful in a questionnaire to help broach the subject:

*"I do think it's an important issue for patients, but obviously for some people it's an embarrassing one to bring up…and it might be that you can't solve the problem…but for some patients it's a very simple 'how many platelets do I need to have sex?"* (Physiotherapist)

All professionals in the focus group preferred the layout of the EORTC tools. This was due to questions having the same response options, making it easier to assimilate information and focus on the problem areas when glancing at the page. There was also consensus that the open question in the POS would be clinically useful, since this would allow patients to highlight any issues of concern that were not specifically asked in the questionnaire.

## Discussion

Myeloma patients are living longer with the complications of their disease and side effects of treatment. With treatment decisions increasingly guided by QOL concerns it is becoming more important to monitor QOL as part of routine clinical care. This is the first study to report a theoretical model of QOL in multiple myeloma, and the first report of the preferences of myeloma patients and clinical staff for the design and clinical utility of QOL questionnaires. These findings provide a platform from which to develop or refine QOL questionnaires for use within the clinical care of myeloma patients.

### Symptoms and QOL

This study helps to characterise the role of symptoms in determining the QOL of myeloma patients. Symptoms were commonly reported by participants as affecting QOL, although only by impacting on other issues (Emotional Status, Activity & Participation, Support Factors). There is clear utility for symptom items in clinical QOL tools, where they might uncover hidden problems and help prioritise or monitor changing symptoms over time. One impact of these findings is that QOL questionnaires should be designed to probe beyond a simple assessment of symptom status (“*What is your current level of pain from 1 to 10?*”), and instead ask for an evaluation of symptom impact (*“How much does pain currently interfere with your life from 1 to 10*?”). This principle is supported by psychometric studies demonstrating that health status and QOL are related but distinct constructs [41,42] – health status alone does not capture all of QOL.

The distinction between ‘health *status*’ and ‘health *evaluation*’ when developing QOL questionnaires has been described previously [43]. Importantly, this difference may affect the suitability of a questionnaire for use in a given setting. For example, the desired utility of a QOL questionnaire in a drug trial may be to identify the prevalence of adverse events (side effects) and detect differences between treatment arms. For this purpose health status questions may be better suited [44]. However, the present study suggests that QOL is better captured by asking respondents to evaluate the impact of symptoms on their life. A recent systematic review identified 13 QOL questionnaires developed or validated for use in myeloma, although most were designed for use in research and focussed on health status, with only a minority asking for health evaluations [13]. This builds a case for further work to refine or develop new QOL tools focussing on health evaluations, which may be better suited to clinical use in people with multiple myeloma. There may also be implications in health economics, where health status questionnaires are the primary tool used in the generation of quality adjusted life years (QALYs) and the allocation of health resources [45], although such tools may not capture all that is important to QOL.

### Health-service issues and QOL

The present study demonstrates the importance of health-service issues in determining the QOL of myeloma patients. Such issues have also been reported as important in other qualitative studies in myeloma [26,27,29,30], making a case for the inclusion of related items in myeloma QOL questionnaires. The EORTC-QLQ-C30 is the most extensively validated QOL questionnaire for use in myeloma [13], but contains no items about health-service issues. Its accompanying myeloma-specific module (the MY24) initially included 4 such items [15], but subsequent psychometric validation resulted in all 4 being removed due to poor psychometric performance (ceiling effects), and the module was revised to the MY20 [14]. The FACT-MM is another myeloma-specific questionnaire that also has no questions about health service factors, though its authors recognise that few patients were involved in its development, and call for more development work using larger patient samples [17].

### Sex and QOL

Sex was identified as important to QOL and impacted by myeloma. Clinical staff identified sex as an important clinical problem, but one that is difficult to discuss with patients. This makes a case for including items about sex in myeloma QOL tools both as an important component of QOL, but also highlights an important clinical utility in facilitating discussion about hidden problems. The EORTC-QLQ-C30 + MY20 together do not contain any items about sex, although this was considered during the development of the myeloma module and is highlighted as an area for future research [15]. The FACT-MM does contain an optional question about the respondent’s satisfaction with their sex life.

### Structure and design of clinical QOL tools

This study highlights a tension in the design of QOL questionnaires, particularly for clinical use. Figure 1 lists 80 QOL issues that would be difficult to cover in 2 sides of A4 (the maximum acceptable length identified here). Acceptability to patients and staff is essential for any clinical tool to be implemented [18,46], yet the need to reduce burden on patients and staff conflicts with the need to be comprehensive. A possible solution would be to combine structured questions and open questions as found in the POS questionnaire [35]. The POS open question was popular amongst both patients and clinical staff, and such questions also resonate with the need for more individualised assessment alongside the use of standard measures [38].

The findings presented here also suggest that the acceptability of questionnaires to respondents and clinical staff may be improved by using the same response options for groups of items. This approach reduces the amount of text for respondents to read and aids the assimilation of information when interpreting completed questionnaires.

### Methodological issues and limitations

One of the limitations of this study is that the majority of sampling took place at large treatment centres in inner London, which may not be representative of the country as a whole. Many patients had however travelled to London from elsewhere in south-east England, and were visiting the London hospitals for their tertiary services. This is a normal service model in the UK, where treatments such as bone marrow transplantation and subsequent follow up are increasingly centralised.

Another sampling limitation is related to the single focus group of clinical staff. Only one such focus group was conducted due to practical challenges taking a multidisciplinary group away from the clinical service at the same time. Further thoughts and ideas may have emerged from additional focus groups of clinical staff.

The use of open questions in the Issues Interviews was designed to elicit the issues most important to patients without introducing bias from the interviewer. However, the interviewer and primary author of the theoretical model (TRO) had an awareness of many existing QOL models at the outset of the study. This could be seen as both a strength and a weakness. This approach allowed the resulting model to build on existing theory, rather than re-inventing the wheel. However, it must be acknowledged that the model is a product of both the patients’ accounts and the researcher’s own expertise and knowledge. The model is an adaptation from Wilson and Cleary [11], but also reflects the work of others. The distinction between activity and participation was drawn from the World Health Organisation’s model of functioning and disability [47], later adapted to incorporate QOL [8]. The role of expectations in determining QOL has been previously described by Calman [7], and the role of adaptation reflects the work of Schwartz and Sprangers on response shift [48]. The role of spirituality and coping mechanisms echoes the work of Zissi into personal characteristics as mediators/determinants of QOL [9].

This study used both individual interviews and focus groups to identify the issues important to QOL, which is reported best practice when establishing content validity of patient reported outcome measures [23]. For the present study it could be argued that the patient focus groups added little to the findings, since they yielded no additional QOL issues beyond those identified in the 20 Issues Interviews. However, the absence of any new themes emerging from the patient focus groups demonstrates that theoretical saturation was reached independently of the chosen interview method (methodological triangulation [32]).

## Conclusions

This study presents the first detailed model of QOL in people with multiple myeloma, alongside their preferences for the design and clinical use of QOL questionnaires. Existing questionnaires may not capture all that is important to QOL from the perspective of people with myeloma, and often miss items on health service factors or sexual function that are important to patients. Questionnaires for use in the clinical care of myeloma patients should capture the *impact* of symptoms on activity, participation or emotional status, and avoid simply asking about symptom status. Clinical questionnaires should not be more than 2 pages long and include open questions to give respondents an individualised voice and focus the goals of care on what is most important to patients. Further work is needed to develop or refine existing QOL tools for use in the clinical care of people with mutiple myeloma.

## Competing interests

The authors declare no competing interests.

## Authors’ contributions

TRO contributed to the study design, acquisition of data, analysis of data and study co-ordination. CR contributed to the acquisition and analysis of data. SdW contributed to the acquisition of data. SAS, RJS, PME and IJH contributed to the conception, design and conduct of the study with IJH acting as senior researcher overseeing the project. TRO prepared the manuscript with all other authors providing comments and critical revisions. The final manuscript was approved by all authors prior to submission.

## Pre-publication history

The pre-publication history for this paper can be accessed here:

http://www.biomedcentral.com/1471-2407/14/496/prepub
